# Increased Myosin light chain 9 expression during Kawasaki disease vasculitis

**DOI:** 10.3389/fimmu.2022.1036672

**Published:** 2023-01-06

**Authors:** Hironobu Kobayashi, Motoko Y. Kimura, Ichita Hasegawa, Eisuke Suganuma, Yuzuru Ikehara, Kazuhiko Azuma, Toshihiro Ito, Ryota Ebata, Yosuke Kurashima, Yohei Kawasaki, Yuki Shiko, Naoki Saito, Hirotaro Iwase, Youngho Lee, Magali Noval Rivas, Moshe Arditi, Masahiko Zuka, Hiromichi Hamada, Toshinori Nakayama

**Affiliations:** ^1^ Department of Immunology, Graduate School of Medicine, Chiba University, Chiba, Japan; ^2^ Department of Pediatrics, Graduate School of Medicine, Chiba University, Chiba, Japan; ^3^ Department of Experimental Immunology, Graduate School of Medicine, Chiba University, Chiba, Japan; ^4^ Chiba University “Synergy Institute for Futuristic Mucosal Vaccine Research and Development (cSIMVa), Japan Initiative for World-leading Vaccine Research and Development Centers, Japan Agency for Medical Research and Development (AMED), Chiba, Japan, Chiba, Japan; ^5^ Division of Infectious Diseases and Immunology, Allergy, Saitama Children’s Medical Center, Saitama, Japan; ^6^ Department of Molecular and Tumor Pathology, Graduate School of Medicine, Chiba University, Chiba, Japan; ^7^ Department of Innovative Medicine, Graduate School of Medicine, Chiba University, Chiba, Japan; ^8^ Clinical Research Center, Chiba University Hospital, Chiba, Japan; ^9^ Department of Legal Medicine, Graduate School of Medicine, Chiba University, Chiba, Japan; ^10^ Department of Pediatrics, Division of Infectious Diseases and Immunology, Guerin Children’s at Cedars-Sinai Medical Center, Los Angeles, CA, United States; ^11^ Infectious and Immunologic Diseases Research Center (IIDRC) and Department of Biomedical Sciences, Cedars-Sinai Medical Center, Los Angeles, CA, United States; ^12^ Department of Forensic Medicine and Pathology, Kanazawa University Graduate School of Medical Sciences, Kanazawa, Japan; ^13^ Japan Agency for Medical Research and Development (AMED)-Core Research for Evolutional Science and Technology (CREST), AMED, Chiba, Japan

**Keywords:** Kawasaki disease, coronary artery, vasculitis, children, platelet, Myl9, CD69

## Abstract

**Introduction:**

Kawasaki disease (KD) is an acute systemic vasculitis that predominantly afflicts children. KD development is known to be associated with an aberrant immune response and abnormal platelet activation, however its etiology is still largely unknown. Myosin light chain 9 (Myl9) is known to regulate cellular contractility of both non-muscle and smooth muscle cells, and can be released from platelets, whereas any relations of Myl9 expression to KD vasculitis have not been examined.

**Methods:**

Plasma Myl9 concentrations in KD patients and children with febrile illness were measured and associated with KD clinical course and prognosis. Myl9 release from platelets in KD patients was also evaluated in vitro. Myl9 expression was determined in coronary arteries from Lactobacillus casei cell wall extract (LCWE)-injected mice that develop experimental KD vasculitis, as well as in cardiac tissues obtained at autopsy from KD patients.

**Results and discussion:**

Plasma Myl9 levels were significantly higher in KD patients during the acute phase compared with healthy controls or patients with other febrile illnesses, declined following IVIG therapy in IVIG-responders but not in non-responders. In vitro, platelets from KD patients released Myl9 independently of thrombin stimulation. In the LCWE-injected mice, Myl9 was detected in cardiac tissue at an early stage before inflammatory cell infiltration was observed. In tissues obtained at autopsy from KD patients, the highest Myl9 expression was observed in thrombi during the acute phase and in the intima and adventitia of coronary arteries during the chronic phase. Thus, our studies show that Myl9 expression is significantly increased during KD vasculitis and that Myl9 levels may be a useful biomarker to estimate inflammation and IVIG responsiveness to KD.

## Introduction

Kawasaki disease (KD) is a pediatric vasculitis that causes inflammation in small and medium-sized arteries and results in the development of coronary artery aneurysms (CAAs) in up to 25% of untreated patients (1). KD is the most common acquired heart disease in children worldwide (2, 3). The standard treatment is high-dose intravenous immunoglobulin (IVIG) which reduces the incidence of CAAs; however, approximately 20% of KD patients are refractory to this treatment (4). Patients with giant aneurysms of >8 mm in diameter or a coronary artery Z score of >10 are at high risk for lifelong cardiac events. The mechanisms of action of IVIG are not entirely understood (5, 6), which emphasizes the need to develop specific and more efficient treatments for KD patients.

Several studies indicate that KD acute inflammation and pathogenesis are linked to systemic cytokine storms (7–9). Their cause is not singular, and it is considered that KD acute inflammation and pathogenesis are linked to aberrant immune responses to infectious disease, vaccination, and burns and cause cytokine storm (10). Genetic studies have identified single-nucleotide variants in several genes associated with increased susceptibility to KD and cardiovascular complications (11, 12). Three of these genes are involved in the calcineurin/Nuclear factor of activated T cells (NFAT) pathway. Combined IVIG therapy with cyclosporin A, which suppresses this pathway, decreases the incidence of CAAs in KD patients predicted to be IVIG resistant (13). Thus, we are beginning to understand the participation of immune cells in KD vasculitis development. However, it still remains unknown why small and medium-sized blood vessels are targeted during KD.

CD69 is a type-II transmembrane protein and a known marker of lymphocyte activation (14, 15). CD69 is also involved in the pathogenesis of inflammatory diseases (14, 16–18). We have previously reported that myosin light chain 9 (Myl9), -12a (Myl12a), and -12b (Myl12b) are functional ligands for CD69. Myl9 is a protein that regulates the cellular contractility of both non-muscle cells and smooth muscle cells. Myl9 can also be released by activated platelets and form net-like structures named “Myl9 nets” inside blood vessels at inflammatory sites (17, 19). We proposed a model called the CD69-Myl9 system in which Myl9 nets likely act as a platform for the recruitment and maintenance of CD69-expressing inflammatory cells in inflamed tissues, thereby exacerbating the inflammation (17). The involvement of the CD69-Myl9 system in airway inflammation and inflammatory bowel diseases has been demonstrated, however, its contribution to KD vasculitis has not been investigated.

The clinical course of KD is closely related to platelet activation (20–23). In the acute phase of KD, the coagulation/fibrinolytic system is enhanced, and platelet counts fluctuate dynamically. KD cases associated with decreased platelet count in the early stage of the disease are considered to be severe cases (24). Furthermore, the Kobayashi risk score system established to identify KD patients at high risk of refractory course considers platelet count among the variables used to predict IVIG-resistance (24). On the other hand, during the convalescence period (2–4 weeks from the first day of illness), the platelet count increases beyond normal levels and then returns to normal (25). Several reports have also shown that the platelet function is enhanced during the first few months after the onset, which is the basis for the duration of the standard treatment with orally administered aspirin (20–23). However, whether platelet activation is involved and contributes to aneurysm formation during KD remains unknown, but thrombotic occlusion is known to account for the sudden death of some KD patients (26).

In this study, we hypothesized that platelets are activated in the acute phase of KD and that Myl9 released by platelets induces inflammatory cell infiltration into coronary artery vessel walls by recruiting CD69-positive cells. We found that plasma Myl9 levels were significantly higher during KD acute phase and that platelets from KD patients showed a preactivated phenotype. The abundant expression of Myl9 was also detected in KD patients’ cardiac tissues obtained at autopsy. Furthermore, using the *Lactobacillus casei* cell wall extract (LCWE) murine model of KD vasculitis, we show that the expression of Myl9 is significantly increased in LCWE-induced cardiovascular lesions. Altogether, our results indicate that Myl9 may be a valuable biomarker to estimate inflammation and IVIG responsiveness to KD.

## Materials and methods

### Patients and autopsy samples

KD patients (n=76), patients with febrile illness (n=16), and healthy volunteers (children; n=8, adult; n=9) included in this study were recruited from April 2010 to March 2021 by the Department of Pediatrics, Chiba University Hospital (Chiba, Japan), and the Department of Pediatrics, Tokyo Women’s Medical University Yachiyo Medical Center (Chiba, Japan) (**Table 1**). We selected patients with febrile illness who required hospitalization and differential diagnosis with KD. Patient information was extracted from the patient’s medical records. The Z scores of the coronary arteries, which are echocardiographic measurements of the internal diameter normalized for body surface area, were calculated by the method of Kobayashi et al. (27), and coronary aneurysms were classified based on the maximum Z score (3). We measured coronary artery size before treatment (at the acute phase, day 0), post-IVIG treatment (day 2-3 after IVIG treatment), at discharge (around day 7 in the case of IVIG-responders and various days depending on the case of each IVIG-non-responder) and in the convalescent phase (around 1 month after onset), and if the coronary artery tends to be dilated, check daily. The maximum Z score in 3 coronary segments of each patient more than 2.5 is considered CAA+.

**Table 1:** Patient information.

	KD (n=76)	Febrile illness (n=16)*	HV (child) (n=8)	HV (adult) (n=9)
Male n (%)	44 (57.9)	8 (50.0)	4 (50.0)	6 (66.7)
Female n (%)	32 (42.1)	8 (50.0)	4 (50.0)	3 (33.3)
Age, months (median [interquartile range])	25 [15-42]	20 [6-66]	36 [18-60]	444 [384-492]
Cases requiring DDx for KD n (%)	76 (100)	6 (37.5)	NA	NA
Admission n (%)	76 (100)	13 (81.3)	NA	NA
ICU care n (%)	3 (3.9)	1 (6.3)	NA	NA
IVIG responder	36 (47.4)	NA	NA	NA
IVIG non responder	40 (52.6)	NA	NA	NA
Patients transferred from other hospitals (IVIG-non responder)	22 (28.9)	NA	NA	NA
CAAs n (%)	24 (31.6)	NA	NA	NA
Z score: ≥2.5, <5	8 (10.5)	NA	NA	NA
Z score: ≥5, <10	10 (13.2)	NA	NA	NA
Z score: ≥10	6 (7.9)	NA	NA	NA
Medication n (%)				
IVIG	76 (100)	0 (0)	NA	NA
ASA	76 (100)	0 (0)	NA	NA
PSL	10 (13.2)	1 (6.3)	NA	NA
mPSL pulse	4 (5.3)	0 (0)	NA	NA
CsA	9 (11.8)	0 (0)	NA	NA
PE	3 (3.9)	0 (0)	NA	NA
DEX	0 (0)	1 (6.3)	NA	NA
ABs	44 (57.9)	7 (43.8)	NA	NA
Death n(%)	0 (0)	0 (0)	NA	NA

*Respiratory infection (n=6), Epstein-Barr virus infection (2), adenovirus infection (1), cervical lymphadenitis (1), Norovirus enteritis (1), Yersinia enteritis (1), urinary tract infection (1), urticaria (1).

HV; Healthy Volunteer, NA; Not applecable, IVIG; Intravenous immunoglobulin, CAAs; Coronary artery abnormalities, ASA; acetylsalicylic acid, PSL; prednisolone, mPSL pulse; intravenous high-dose methylprednisolone pulse therapy, CsA; cyclosporin A, PE; plasma exchange, DEX; dexamethasone, Abs; antibiotics.

Plasma samples for Myl9 measurement were collected from 76 KD patients in the acute phase (before IVIG treatment, day 0), post-IVIG treatment (day 2-3 after IVIG treatment), at discharge (around day 7 in the case of IVIG-responders and various days depending on the case of each IVIG-non-responder) and in the convalescent phase (around 1 month after onset). Twenty-two of 76 KD patients were transferred from other hospitals due to a lack of response to IVIG treatment and/or CAAs, and thus lacked plasma samples from before IVIG treatment. Patients were defined as IVIG non-responders when their body temperature was ≥37.5°C at 48 hours after the initiation of IVIG treatment or when they had a relapse of KD, defined as a return of fever (≥37.5°C) after a ≥48-hour afebrile period from the 1^st^ IVIG treatment and needed additional therapies. Patients with febrile illness included those with a respiratory infection (n=6) or with other febrile illnesses in whom a diagnosis other than KD was confirmed, such as Epstein-Barr virus infection (2), adenovirus infection (1), cervical lymphadenitis (1), norovirus enteritis (1), *Yersinia* enteritis (1), urinary tract infection (1), and urticaria (1).

Platelet-rich plasma samples were collected from 9 KD patients and 5 healthy volunteers. All 9 cases were 1^st^ IVIG responders, and all samples were collected before IVIG treatment.

Peripheral blood mononuclear cells (PBMCs) were collected from 6 KD patients. All 6 cases were 1^st^ IVIG responders, and samples were collected in the acute phase (pre-IVIG) and convalescent phase (1 month after KD onset).

Three autopsy specimens from KD patients with coronary artery aneurysms and one from a control child who died from trauma were collected from the Department of Forensic Medicine, Graduate School of Medicine, Chiba University, and the Department of Forensic Medicine, Graduate School of Medicine, Kanazawa University (Kanazawa, Japan) (**Table 2**). The diagnosis of each KD patient was confirmed based on clinical features after the exclusion of other inflammatory diseases and based on the cardiac tissue findings.

**Table 2:** Autopsy samples.

	No.	Sex	Age at death	Cause of death	Time from KD onset to death
KD	1	Male	3 months	AMI	14 days
	2	Female	8 years	AMI	1 year
	3	Female	2 years	AMI	2 years
control	1	Female	1 year	trauma	

AMI; acute myocardial infraction.

The study was approved by the Ethics Committee of Chiba University Graduate School of Medicine (No. 3683). Written informed consent was provided by all participants or their legal guardians (for children who could not give consent) before blood sampling.

### Mice

C57BL/6 male mice were purchased from CLEA Co. (Tokyo, Japan). They were maintained under specific pathogen-free conditions and were used at 5 weeks of age. All animal experiments were approved by the Chiba University Review Board for Animal Care.

### LCWE-induced KD vasculitis murine model

*Lactobacillus casei* (ATCC 11578) cell wall extract was prepared as previously reported (28, 29). Five-week-old male mice were intraperitoneally injected with 500 μg of LCWE. Three days, 7 days or 14 days later, the mice were euthanized, and hearts were removed and embedded in the Optimal Cutting Temperature (OCT) compound for a histological examination.

### Tissue fixation, staining, and histopathology (murine sample)

Serial cryosections (10 μm) of heart tissue were fixed in ethanol and formalin, then stained with hematoxylin–eosin (HE). All images were acquired with a BZ-X710 microscope (Keyence, Osaka, Japan). Histopathological scoring of coronary arteritis was performed with the following scoring system: Score 0 = no inflammation, 1 = rare inflammatory cells, 2 = scattered inflammatory cells, 3 = diffuse infiltration of inflammatory cells, and 4 = dense clusters of inflammatory cells. Scoring was performed on each 100 μm of the five consecutive sections.

For immunohistochemistry (IHC) analysis, heart cryostat sections (10 μm) were fixed in 4% paraformaldehyde and then stained with goat anti-CD69 (R & D Systems, Minneapolis, MN) overnight at 4°C, rabbit anti-Myl9/12 (F6; Abwiz Bio, San Diego, CA), mouse Cy3-conjugated anti-α−SMA (alpha-smooth muscle actin) (1A4; Sigma-Aldrich, St. Louis, MO) for 2 h at room temperature. Isotype controls (normal goat IgG (R & D Systems) and normal rabbit IgG (Bio X cells, Lebanon, NH)) were used as negative controls. After washing, the sections were incubated with Alexa Fluor 488-conjugated anti-rabbit IgG and Alexa Fluor 647-conjugated anti-goat IgG (Thermo Fisher Scientific, Waltham, MA) for 1 h as secondary antibodies. All images were acquired with an LSM 710 (Carl Zeiss, Oberkochen, Germany), and data sets were analyzed with the ImageJ software program (National Institutes of Health, Bethesda, MD, USA) (30).

### Tissue fixation, staining, and histopathology (human sample)

Human autopsy specimens were fixed in formalin and embedded in paraffin. Sections were then prepared from paraffin-embedded materials and subjected to HE staining, Elastica-van Gieson (EVG) staining, and IHC staining using appropriate antibodies (anti-Myl9/12 antibody, anti-smooth muscle actin antibody).

### Preparation of platelet-rich plasma and thrombin stimulation

Platelet-rich plasma was prepared from peripheral blood, as previously described (31). In brief, 1 ml of blood was collected with a sodium citrate buffer tube and added to 5 ml of PIPES, and the solution was spun at 100 × *g* for 15 min. The platelet-rich supernatant was collected, and 1 U/ml apyrase and 1 μM PGE1 (final concentrations) were added, then the tubes were spun at 1000 × *g* for 10 min. The platelet pellet was resuspended in 200 μl of Tyrode’s buffer and then divided into two equal parts. One was incubated with 100 mU/ml thrombin and 1 mM CaCl_2_ for 2 hours at 37°C for activation. The other was stored at 4°C for the same time without stimulation. After 2 hours of incubation, they were spun at 15000 rpm for 10 min, and the supernatants were collected to measure Myl9 level.

### Measurement of the Myl9 level by enzyme-linked immunosorbent assay

Myl9 levels in human plasma and Myl9 released from platelets in *in vitro* culture were measured by enzyme-linked immunosorbent assay (ELISA; Human MLC2/MYL9 ELISA Kit, LifeSpan BioSciences, Seattle, WA, USA) according to the manufacturer’s instructions.

### Flow cytometry

For human samples, PBMCs were isolated from EDTA blood samples by Ficoll density gradient centrifugation (Ficoll-Paque PLUS, GE Healthcare, Chicago, IL) and stored with 1 ml of LaboBanker (TOSC, Tokyo, Japan) at -20°C. The cryopreserved PBMCs were thawed, washed 2 times with saline, and then suspended in PBS-BSA containing 0.1% NaN_3_. The cells were stained with fluorochrome-conjugated antibodies with the following specificities: human CD69 (FN50; BioLegend, San Diego, CA), human CD3 (UCHT1; BioLegend), human CD4 (A161A1; BioLegend), human CD8 (RPA-T8; BioLegend), human CD14 (61D3; Thermo Fisher Scientific), human CD16 (3G8; BioLegend), human CD19 (HIB19; BD Biosciences, Franklin Lakes, NJ), for 40 min at 4°C in the dark, washed with PBS-BSA containing 0.1% NaN_3._ Cellular viability was determined using propidium iodide (PI). Frequencies of lymphocytes (CD4^+^ T cells, CD8^+^ T cells, and B cells) and frequencies of NK cells and monocytes were assessed with two separate flow staining panels. Cells were analyzed with a Canto II flow cytometer (BD Biosciences).

For murine samples, 100μl of peripheral blood was first lysed with ACK buffer (Thermo Fisher Scientific), and then stained with fluorochrome-conjugated antibodies with the following specificities: mouse CD69 (H1.2F3; BioLegend), mouse Ly-6G (1A8; BioLegend), mouse Ly-6C (HK1.4; BioLegend), mouse CD3ϵ (145-2C11; BioLegend), mouse CD11b (M1/70; BioLegend), mosue NK-1.1 (PK136; BioLegend), mouse CD45R/B220 (RA3-6B2; BD Biosciences), 7-AAD Viability Staining Solution (PK136; BioLegend) for 40 min at 4°C in the dark, washed with PBS-BSA containing 0.1% NaN_3_ and then analyzed with a Fortessa flow cytometer (BD Biosciences). The data were analyzed using the FlowJo software program (BD Biosciences).

### Statistical analysis

All statistical analyses were performed with the GraphPad Prism software program (GraphPad, San Diego, CA). Statistical significance was determined using a Mann-Whitney *U* test or Wilcoxon signed-rank test for comparisons between two groups. A one-way analysis of variance (ANOVA) was used for multiple comparisons. Pearson’s correlation coefficient was used to analyze the correlation between two parameters. P values of < 0.05 were considered to indicate statistical significance.

## Results

### Plasma Myl9 levels are elevated in acute-phase KD patients and may reflect the disease activity in KD as measured by inflammatory markers

**Figure 1:**
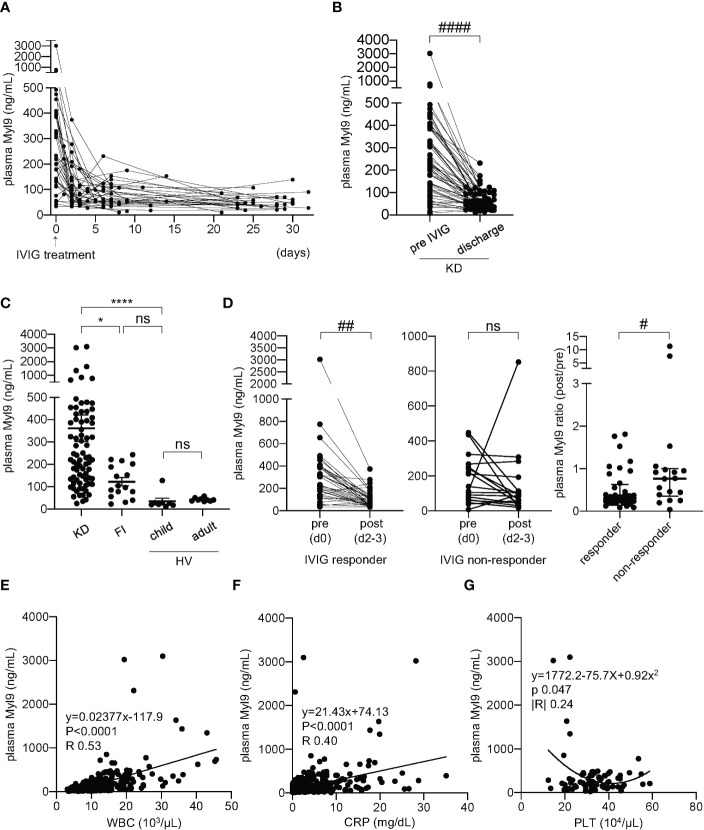
Plasma Myl9 levels correlate with KD clinical course. **(A)** A time-course analysis of plasma Myl9 levels in 36 KD patients who responded to IVIG treatment. Day 0 values are values of samples obtained just before IVIG treatment, and the same patient samples are connected by a line. **(B)** Comparison of the plasma Myl9 levels in the acute phase (pre-IVIG and at discharge of KD patients (n=54). The same patient samples are connected by a line. ####*P* < 0.0001 (single comparisons, Wilcoxon signed-rank test without correction). **(C)** Comparison of the plasma Myl9 levels in the acute phase of KD patients (n=76), patients with febrile illness (FI) (n=16), child healthy volunteers (HV) (n=8), and adult HV (n=9). ****P < 0.0001 (multiple comparisons, one-way ANOVA), *P < 0.05. ns, not significant. Data are shown as the mean ± SEM. **(D)** Myl9 levels of KD patients before (day 0) and after (day 2-3) IVIG treatment, including both IVIG responders (n=36) (left) and non-responders (n=18) (middle). Myl9 level ratio (post/pre-IVIG) of IVIG responders (n=36) and non-responders (n=18) (right). The same patient samples are connected by a line. Plasma Myl9 levels decreased significantly after IVIG treatment in the IVIG responder group. ##*P* < 0.01 (single comparisons, Wilcoxon signed-rank test without correction), #*P* < 0.05. ns, not significant. Data are shown as the median with interquartile range. **(E, F)** The relationship between plasma Myl9 levels and the white blood cell (WBC) counts **(D)**, or CRP levels **(E)** (n=415). Plots show samples from 76 KD patients in the acute to convalescent phase. **(G)** The relationship between the maximum plasma Myl9 levels and the minimum platelet (PLT) counts in each KD patient (n=76).

To investigate the potential connection between Myl9 and KD clinical course, we first quantified Myl9 concentrations in the plasma of KD patients over time. We selected KD patients who underwent IVIG treatment immediately after diagnosis and who responded to IVIG treatment. Plasma Myl9 levels were significantly high during KD acute phase, just before IVIG treatment (day 0), and decreased over time (**Figure 1A**). Notably, plasma Myl9 levels were significantly higher during acute KD when compared with convalescent KD patients at the time of discharge (**Figure 1B**). We next determined if elevated plasma Myl9 levels observed in KD patients were specific to KD. To this end, we quantified Myl9 levels in the plasma of patients with other febrile illnesses in whom a diagnosis other than KD was confirmed. We found that plasma levels of Myl9 were significantly higher in KD patients when compared with healthy volunteers (HV, children, and adults) and patients with other febrile illnesses (FI) (**Figure 1C**). On the other hand, there was no significant difference between plasma Myl9 levels of febrile patients and healthy children (**Figure 1C**). Interestingly, plasma Myl9 levels significantly decreased after the administration of IVIG in the IVIG responder group (**Figure 1D**, left panel), whereas no difference was observed in Myl9 levels before and after administration of IVIG in IVIG non-responder KD patients (**Figure 1D**, middle panel). Notably, the plasma Myl9 ratio post/pre-treatment in IVIG responder patients was less than 1, indicating that Myl9 levels decreased upon treatment. In contrast, in IVIG non-responder patients, this ratio was significantly higher, indicating that their plasma Myl9 levels are not reduced upon treatment (**Figure 1D**, right panel). We next investigated the relationship between plasma Myl9 levels and the laboratory characteristics of KD patients. Plasma Myl9 levels showed a significant correlation with markers of inflammation, such as white blood cell (WBC) counts and C-reactive protein (CRP) levels, which are known to be positively correlated with KD disease activity (**Figures 1E, F**). Furthermore, acute-phase plasma Myl9 levels in KD patients had a negative correlation with the minimum platelet counts (**Figure 1G**), which are known to correlate with inflammation (24, 32). Our results suggest that plasma Myl9 concentrations may reflect the inflammatory status during acute KD.

CAAs represent the most serious complication of KD. Thus, we examined whether plasma Myl9 levels in the acute phase were associated with disease outcomes. There was no significant difference in plasma Myl9 levels before the first IVIG treatment between IVIG responders and non-responders (**Supplemental Figure 1A**), between patients with or without CAAs (**Supplemental Figure 1B**), or between patients with or without CAAs among IVIG non-responders (**Supplemental Figure 1C**). However, interestingly, all four patients with high Myl9 levels of >1000 ng/ml developed moderate or giant CAAs with a Z score of >5 **(Supplemental Figures 1D, E**), and there was a weak positive correlation between the plasma Myl9 levels and Z scores (**Supplemental Figure 1D**), although there was no significant difference in the plasma Myl9 level before IVIG treatment between patients with (Z score ≥ 2.5) and without CAAs (**Supplemental Figure 1E**). These data suggest that very high plasma Myl9 levels of >1000 ng/ml during KD acute phase may indicate increased risk of CAA development.

### Platelet abnormality in KD patients

**Figure 2:**
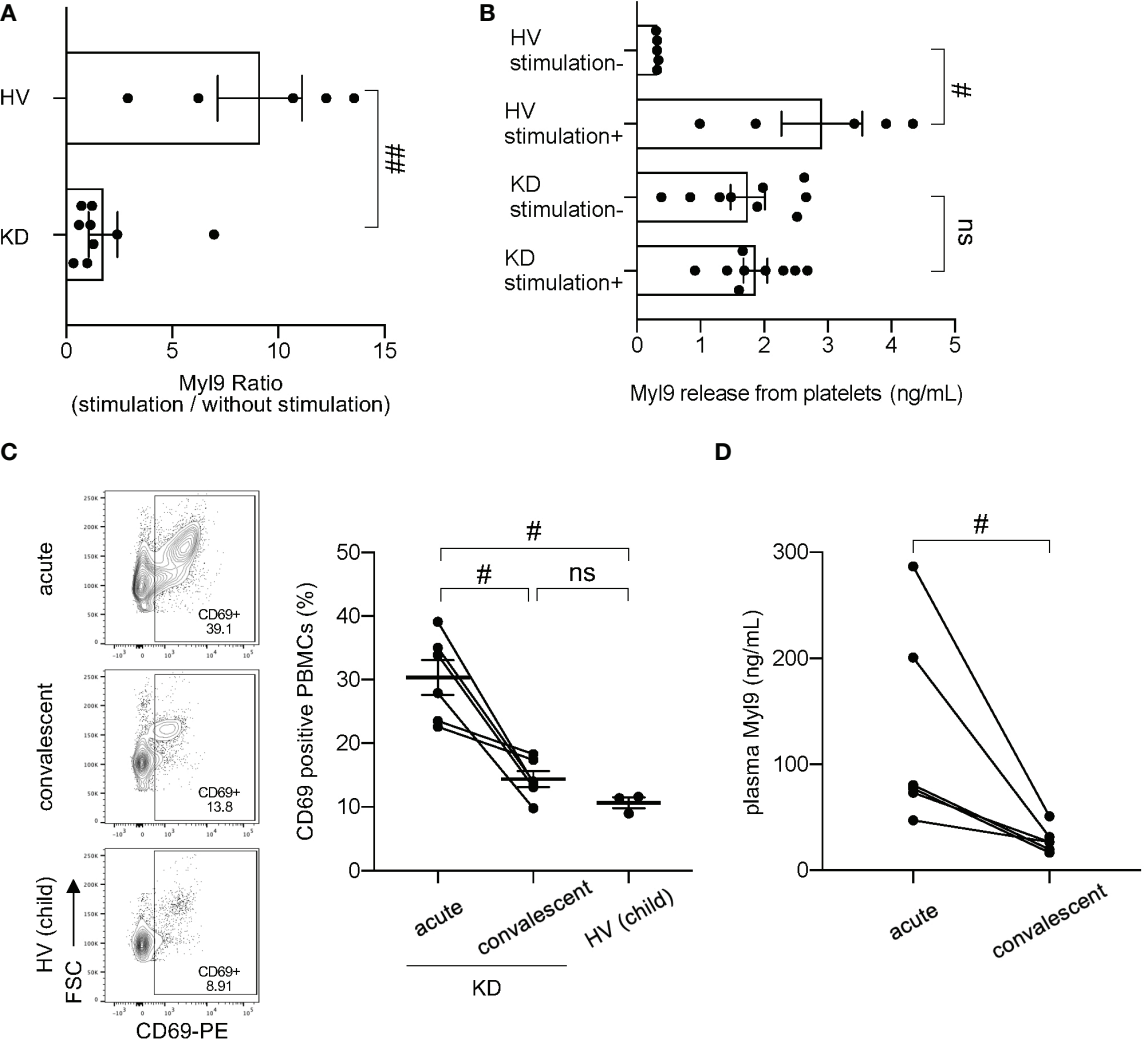
Myl9 and CD69 expression in peripheral blood mononuclear cells (PBMCs) in KD patients. **(A)** Myl9 release from platelets of KD patients (n=9) in the acute phase of the disease in comparison to HV (n=5), with or without *in vitro* stimulation with thrombin. The graph indicates the ratio of the Myl9 levels released with stimulation to those released without stimulation. ##*P* < 0.01 (single comparisons, Mann-Whitney *U* test without correction). Data are shown as the mean ± SEM. **(B)** Myl9 release from platelets of KD patients (n=9) in the acute phase of the disease and HV (n=5), with or without *in vitro* stimulation with thrombin. #*P* < 0.05 (single comparisons, Mann-Whitney *U* test without correction). Data are shown as the mean ± SEM. **(C)** Flow cytometric analysis showing CD69-expressing PBMCs in a KD patient (IVIG responder) in the acute phase, in the convalescent phase (1 month after IVIG treatment), and in a child HV. The right graph shows the percentage of CD69-positive PBMCs of KD patients in the acute and convalescent phase (n=6) and HV (n=3). The same patient samples are connected by a line. #*P* < 0.05 (single comparisons, Wilcoxon signed-rank test without correction). ns, not significant. Data are shown as the mean ± SEM. **(D)** Plasma Myl9 levels of KD patients in the acute phase and convalescent phase (n=6). These samples were taken from the patients at the same time as the analysis shown in **(C)** #*P* < 0.05 (single comparisons, Wilcoxon signed-rank test without correction).

Since Myl9 can be released from activated platelets (14, 17) and platelets from KD patients appear abnormal, as shown by their enhanced capacity to aggregate and their increased release of platelet-derived microparticles (21–23), we next examined the ability of platelets from KD patients to release Myl9. We collected platelet-rich plasma from both KD patients and healthy volunteers, either unstimulated or stimulated with thrombin *in vitro*, and measured the amounts of Myl9 in the supernatant during *in vitro* culture. The ratio of the Myl9 concentration with thrombin stimulation to that without stimulation was calculated (**Figure 2A**). Myl9 ratio was significantly higher in the supernatant from healthy volunteers’ platelets, demonstrating that healthy platelets can release Myl9 upon stimulation. However, this ratio was decreased in cultures with platelets from KD patients, indicating that platelets from KD patients did not respond to *in vitro* thrombin stimulation. Further, Myl9 was significantly released from healthy volunteers’ platelets upon stimulation, not from platelets in KD patients (**Figure 2B**). Notably, Myl9 levels released from platelets without stimulation were significantly higher in KD patients, suggesting that platelets from KD patients appeared to be pre-activated. Since plasma Myl9 levels were significantly higher during KD acute phase, it is possible that platelets from KD patients are pre-activated *in vivo* and, therefore unable to further respond to thrombin activation *in vitro*.

### Increased CD69-positive cells in whole blood of KD patients

We next determined the expression of CD69-expressing cells in the peripheral blood of KD patients. Compared with HV, we observed a significant increase in CD69-positive cells in peripheral blood of KD patients during the acute phase of the disease (30.33% ± 2.73%) (**Figure 2C**). The percentage of CD69-positive cells in the convalescent phase (1 month after KD onset) (14.40% ± 1.26%) was comparable to the one observed in healthy children (10.66% ± 0.84%; mean ± SEM) (**Figure 2C**). Several types of immune cells, including CD4^+^ T cells, CD8^+^ T cells, B cells and NK cells upregulated CD69 during the acute phase of KD, whereas classical and non-classical/intermediate monocytes already expressed CD69 at steady-state (**Supplemental Figures 2A–C**). However, we did not observe differences in numbers of total CD69 positive cells between KD acute phase and convalescent phases (**Supplemental Figure 3A**). In our experiments, we collected PBMCs from our patients, which contain lymphocytes and monocytes, but no granulocytes such as neutrophils. It has been known that the number of neutrophils in the blood increases tremendously during the acute phase of KD, while the number of lymphocytes decreases. In fact, our data showed that total white blood cell (WBC) counts, and neutrophil (Neu) counts were high during the acute phase and dramatically decrease in KD convalescent phase; whereas lymphocyte (Lym) counts were low at the acute phase and dramatically recovered at the convalescent phase (**Supplemental Figure 3B**). Therefore, we think that the reason why the number of CD69 positive PBMCs did not change between KD acute and convalescent phase is due to the decreased number of lymphocytes during the acute phase. We also confirmed that plasma Myl9 levels from the patients used in **Figure 2C** were elevated in the acute phase and significantly decreased in the convalescent phase after IVIG treatment (**Figure 2D**). These data suggest a potential involvement of the CD69-Myl9 system in the pathogenesis of KD.

### Increased expression of Myl9/12 in inflamed coronary arteries during LCWE-induced KD vasculitis

**Figure 3:**
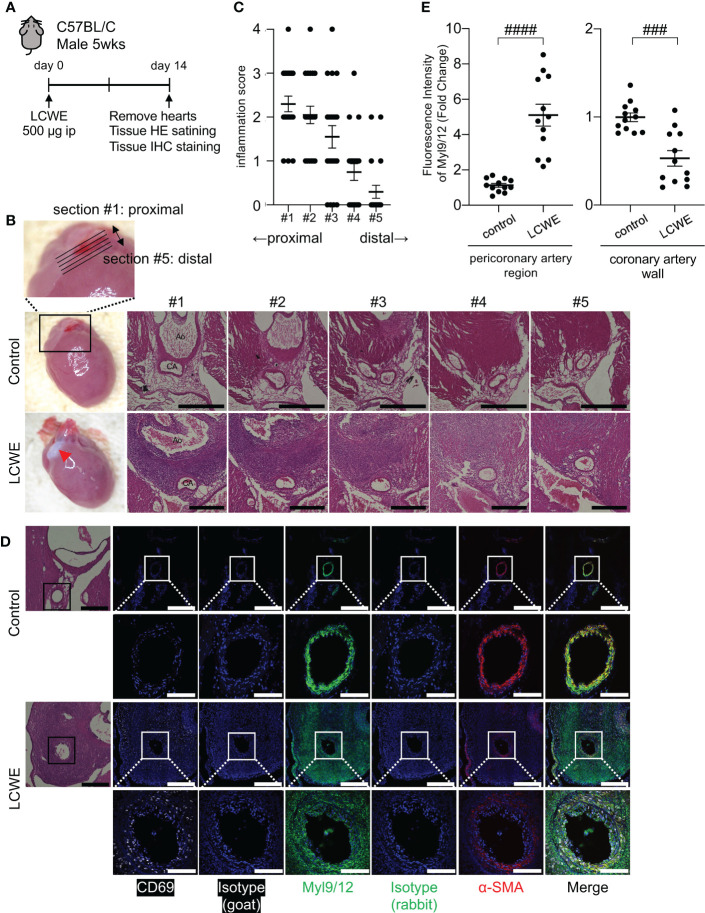
Myl9/12 expression in coronary arteries of LCWE-injected mice. **(A)** Schematic outline of the LCWE-induced KD vasculitis murine model. Five-week-old WT male mice were intraperitoneally (i.p.) injected with 500µg of LCWE and 14 days later heart tissues were collected and analyzed. **(B)** Representative pictures of heart tissues collected from control mice and LCWE-injected mice developing vasculitis (left). HE staining of serial sections of coronary arteries of a control mouse (upper) and a LCWE-injected mouse (lower). Five consecutive sections were prepared at 100 μm intervals from the proximal side of the coronary artery origin (section #1) to the distal side (section #5) as shown in the enlarged photograph (upper). Red triangle indicates inflammatory change. Black scale bars indicate 500 μm. CA, coronary artery; Ao, aorta. **(C)** The inflammation scores of serial sections of coronary arteries of the LCWE-injected mice in B are shown (100 tissue sections from 20 mice). Each dot shows the inflammation score from each section. **(D)** HE staining (left) and IHC staining of the coronary artery in a control mouse (upper) and an LCWE-injected mouse (lower) using goat anti-CD69 Ab (white), goat IgG isotype Ab, rabbit anti-Myl9/12 Ab (green), rabbit IgG isotype Ab, and anti-α-SMA Ab (red). The coronary artery is surrounded by a black (HE) or white square (IHC), and the indicated area is shown in a high-power field (lower). Scale bars indicate 500 μm (low-power field) and 100 μm (high-power field). **(E)** The fluorescence intensity of Myl9/12 in the pericoronary artery region (left) and coronary artery wall (right) using the section #1, comparison between control mice (n=12), and LCWE-injected mice (n=12), normalized to isotype controls was shown. Data are shown as the mean ± SEM. ####*P* < 0.0001 (single comparisons, Mann-Whitney *U* test without correction), ###*P* < 0.001. Data are shown as the mean ± SEM.

We used the LCWE-induced murine model of KD vasculitis to assess Myl9 expression in cardiovascular lesions (28). In this model, 5 weeks old male wild-type (WT) mice are intraperitoneally injected with LCWE, and heart tissues are collected and analyzed 14 days post-LCWE injection (**Figure 3A**). Macroscopically, inflammatory changes were observed at the base of the heart in LCWE-injected mice (**Figure 3B**, left). Compared with control mice, LCWE injection resulted in massive inflammatory cell infiltrations around the coronary arteries (**Figure 3B**).

To determine the spread of inflammation of the coronary artery, we prepared five consecutive tissue sections spanning the coronary artery, as shown in **Figure 3B**, from the most proximal (section #1) to the most distal part (section #5). Inflammation was more prominent on the proximal side of coronary artery (section #1) than the distal side (section #5) (**Figures 3B, C**). Immunohistochemistry (IHC) staining of heart tissue sections revealed that many inflammatory cells infiltrating the coronary artery after LCWE injection expressed CD69 (**Figure 3D**, left). In addition, the intensity of α-smooth muscle cell actin (α-SMA), a contractile protein present in vascular smooth muscle cells (VSMCs), was significantly decreased in the inflamed coronary arteries 14 days after the administration of LCWE (**Figure 3D**, right). We next examined Myl9 expression using an antibody that detects Myl9, Myl12a and Myl12b (17). Interestingly, expression of Myl9/12 was detected in the coronary artery in both control and LCWE-injected mice (**Figure 3D**). However, the expression pattern was quite different. In LCWE-injected mice, Myl9/12 expression was broadly spread around the inflamed coronary artery, where massive cellular infiltrations are observed (**Figure 3D**). On the other hand, in healthy control mice, Myl9/12 expression completely overlapped with the expression pattern of α-SMA in the coronary artery wall (**Figure 3D**). Quantification analysis revealed that the intensity of Myl9 expression in the pericoronary artery region was significantly higher in LCWE-injected mice than in control mice. In contrast, the intensity of Myl9 expression in the coronary wall was decreased in LCWE-injected mice than in control mice (**Figure 3E**).

### Myl9/12 expression is detected in coronary artery aneurysms ahead of inflammation

**Figure 4:**
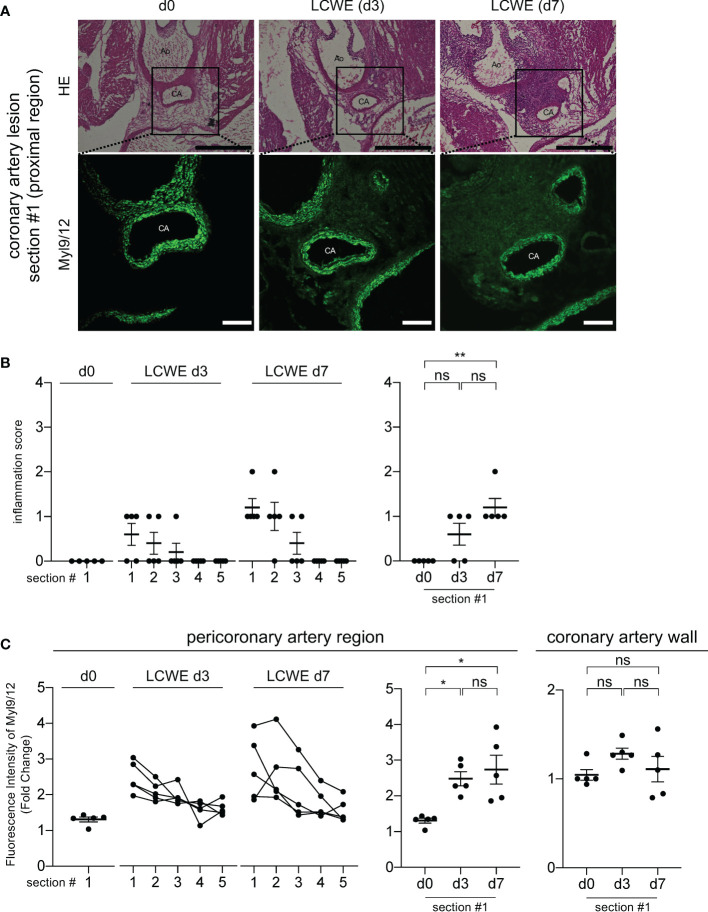
Myl9/12 is detected in coronary arteries of LCWE-injected mice prior to the infiltration of inflammatory cells. **(A)** HE staining (upper) and IHC staining using anti-Myl9/12 Ab (lower) of the proximal side (section #1 indicated in **Figure 3B**) of the coronary artery in a control mouse (left) and an LCWE-injected mouse at 3 days (middle) and 7 days (right) after the administration of LCWE. Scale bars indicate 500 μm (low-power field) and 100 μm (high-power field). CA, coronary artery; Ao, aorta. **(B)** Inflammation scores of five consecutive sections of the coronary arteries of LCWE-injected mice on day 0 (d0, n=5), day 3 (LCWE d3, n=5), and day 7 (LCWE d7, n=5) after the administration of LCWE. Section #1 is the most proximal side, and section #5 is the most distal side of the origin of the coronary artery (left). The inflammation scores using section#1 compared among d0, d3 and d7 was shown (right). **P < 0.01 (multiple comparisons, one-way ANOVA), ns; not significant. Data are shown as the mean ± SEM. **(C)** The fluorescence intensity of Myl9/12 in the pericoronary artery region (left and middle) and in the coronary artery wall (right) among control mice (d0, n=5), and LCWE-injected mice on day 3 (LCWE d3, n=5) and day 7 (LCWE d7, n=5) after the administration of LCWE. Section #1 is the most proximal side and #5 is the most distal side (left). The fluorescence intensity of Myl9/12 in the peri-coronary artery (middle) and in the coronary artery wall (right) using the section #1 compared among d0, d3 and d7 was shown. Data were normalized to controls. *P < 0.05 (multiple comparisons, one-way ANOVA). ns, not significant. Data are shown as the mean ± SEM.

Myl9/12 is highly detected in the tunica media of healthy mice and its expression decreased in heart tissue sections from LCWE-injected mice. These observations raised the question of the source of Myl9 in inflamed coronary arteries during LCWE-induced KD vasculitis, which could either be activated platelets or VSMCs. Therefore, we next examined heart sections from LCWE-injected mice at baseline, 3 days and 7 days after administration of LCWE (**Figures 4A–C**). We observed little inflammatory cell infiltrations on day 3 post-LCWE-injection, and the inflammation score at baseline (day 0) and 3 days post-LCWE injection were not significantly different, even in the most proximal part of the coronary artery (section #1) where a prominent inflammation is observed at day 14 post-LCWE injection (**Figures 3C**, **4B**, right). Compared to baseline (day 0), the inflammation score was slightly but significantly elevated at 7 days post-LCWE-injection (**Figure 4B**, right). However, the degree of inflammation was still much lower than the one observed on day 14 post-LCWE-injection (**Figure 3C**). We next examined Myl9 expression levels separately in the coronary artery wall and pericoronary artery region where massive inflammatory cell infiltrations were observed on day 14 post-LCWE-injection. Notably, Myl9/12 expression in the pericoronary artery region was already detected on day 3, particularly on the proximal side of the origin of the coronary artery (section #1), and the intensity of Myl9/12 in the pericoronary artery region was significantly higher on day 3 than on day 0 (**Figure 4C**, middle). On the other hand, Myl9 intensity in the coronary artery wall did not change over time (**Figure 4C**, right). Thus, the expression of Myl9/12 was detected on day 3 post-LCWE injection, when inflammatory cell infiltrations were not yet observed by HE staining and severe vasculitis was not yet induced.

We next examined which circulating cellular subsets express CD69 in LCWE-injected mice (**Supplemental Figure 4**). We found that the frequencies and absolute numbers of NK cells, neutrophils, eosinophils and monocytes were increased in the blood of LCWE-injected mice when compared with control mice (**Supplemental Figures 4A, B**). We also observed increased frequencies of circulating NK cells expressing CD69 in LCWE-injected mice (**Supplemental Figure 4C**, left) and our analysis further revealed that absolute numbers of circulating NK cells, neutrophils, eosinophils and Ly6c^neg^ monocytes expressing CD69 were significantly increased in LCWE-injected mice (**Supplemental Figures 4B, C**, right). In conjunction with our results showing increased expression of CD69 by circulating NK cells, T and B cells in KD patients (**Supplemental Figure 2**), these data indicate that various kinds of immune cells could be activated and infiltrate the vascular walls during KD pathogenesis.

### Myl9/12 is detected in coronary arteries from KD patients

**Figure 5:**
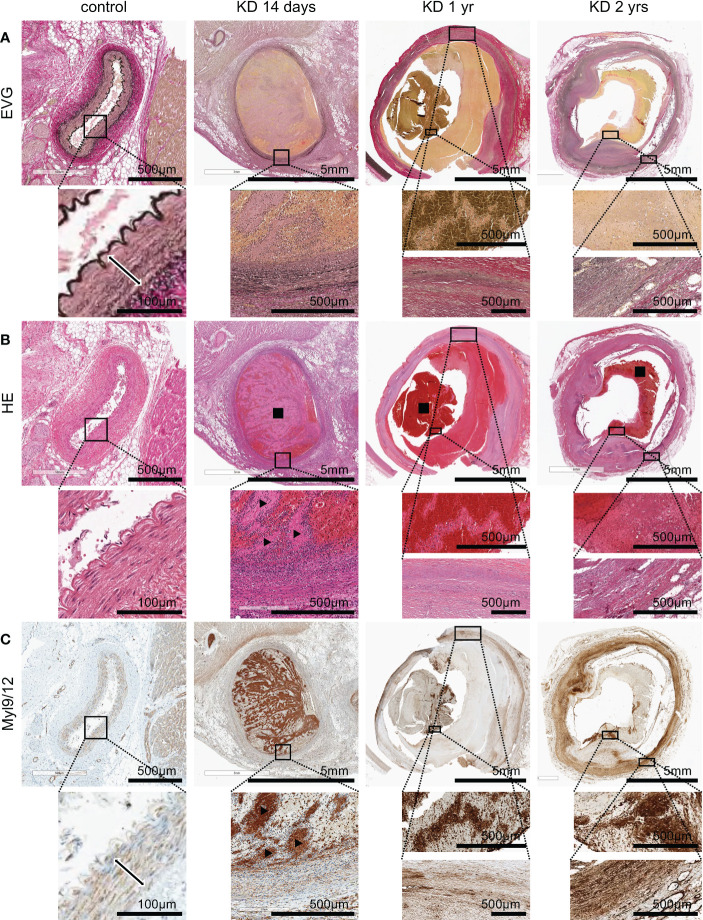
Myl9/12 expression in heart tissues collected from KD patients. **(A)** Elastica-van Gieson (EVG) staining of the coronary artery in a control patient (left), KD patients who died on the 14th day of illness (2nd from left), at 1 year after illness (3rd from left), and 2 years after illness (right). Each upper figure shows the coronary artery (giant coronary artery aneurysm in KD patients), and the area surrounded by the square indicates a high-power field. The double-headed arrow indicates medial smooth muscle in the control patient. **(B)** HE staining of the same area shown in **(A)** Black square indicates thrombus, black arrowhead indicates white thrombus. **(C)** Myl9/12 staining of the same area shown in **(A, B)** Double-headed arrow indicates medial smooth muscle. Black arrowhead indicates white thrombus.

We next examined whether Myl9/12 expression was also detected in the coronary arteries of KD patients. We obtained three tissue samples from autopsied KD patients: one sample from the acute phase (14 days of illness) and two samples from the chronic phase (1 year and 2 years after KD, respectively), and performed Elastica-van Gieson (EVG) staining (**Figure 5A**), HE staining (**Figure 5B**) as well as anti-Myl9/12 staining (**Figure 5C**).

In heart tissue sections from a control patient, the three-layer structure of the coronary artery was maintained (**Figure 5A**, left), and no inflammatory cells were detected (**Figure 5B**, left). Myl9/12 was only detected in the media, and absent in the intima and adventitia as well as outside the adventitia (**Figure 5C**, left).

EVG staining of a heart tissue section collected during KD acute phase (a patient on day 14 of illness) revealed that the three-layer structure of the coronary artery had already been destroyed (**Figure 5A**). HE staining showed massive cell infiltration from inside the thrombus to the vessel wall with infiltrating cells inside the thrombus, especially surrounding the white thrombus (**Figure 5B**). Importantly, Myl9/12 was most strongly expressed in thrombus, especially in white thrombus, and was then strongly expressed in the intima side of the vessel wall and adventitia-side tissues (**Figure 5C**).

In the chronic phase, at 1 year and 2 years post-KD, EVG staining revealed that the three-layer structure of the coronary artery had been destroyed in both cases (**Figure 5A**), although we only detected a few infiltrating cells (**Figure 5B**), suggesting that inflammation already disappeared. The expression of Myl9/12 in the thrombus became weaker, whereas the expression of Myl9/12 on the blood vessel walls was relatively strong.

## Discussion

In the present study, we demonstrated that Myl9 expression levels were significantly high in the peripheral blood of KD patients during the acute phase of the disease and that Myl9/12 expression was elevated in inflamed coronary arteries from both KD patients and LCWE-injected mice. Notably, plasma Myl9 levels were well correlated with KD inflammatory status, as measured by inflammatory markers such as CRP, and significantly higher in KD patients than in patients with other febrile pediatric illnesses. Furthermore, platelets collected from KD patients in the acute phase of the disease appeared to be activated. These data suggest that plasma Myl9 levels may be a marker for KD inflammation.

The involvement of Myl9 released from activated platelets in inflammation was first discovered by our group in 2016 (17). We have proposed a model named the CD69-Myl9 system, wherein CD69-expressing inflammatory leukocytes utilize ‘Myl9 nets’ to migrate into and remain within inflamed tissues, exacerbating inflammation (14). The CD69-Myl9 system is reported to be involved in the pathogenesis of airway inflammation, polyp formation of eosinophilic rhinosinusitis (ECRS) patients, and inflammatory bowel diseases, such as ulcerative colitis and Crohn’s disease (17, 18). Since activated platelets release Myl9, it has been suggested that the CD69-Myl9 system is involved in various inflammatory diseases, including vasculitis (14). In this study, we discovered that Myl9 was highly detected in inflamed coronary arteries in LCWE-injected mice and heart tissues from KD patients collected during autopsy. In addition, we found numerous CD69-positive inflammatory cells on and around the inflamed vessel wall of LCWE-injected mice, where abundant Myl9 was detected. We observed that several immune cells (NK cells, T cells, and B cells) upregulated CD69 expression during KD acute phase. Our analysis also indicates that monocytes constitutively expressed CD69. Thus, the elevated frequency of CD69-positive cells in PBMCs from KD patients is not only due to CD69 upregulation by lymphocytes, and monocytes may potentially contribute to this process, especially during the acute phase of the disease. Indeed, Ikeda et al. also showed increased CD69-positive monocytes in the peripheral blood during the acute phase of KD, which returns to normal levels during the convalescent phase (33). While we observed only low frequencies of circulating CD69-expressing cells in the blood of LCWE-injected mice, we did find significant increases in CD69-positive NK cells in both LCWE-injected mice and in PBMCs at the acute-phase of KD patients. Thus, our results indicate a possible involvement of the CD69-Myl9 system in the pathogenesis of KD vasculitis, although further experiments are required to show the functional contribution of this system to KD vasculitis.

We previously reported that platelet activation promotes the release of Myl9 (17), and it has been reported that platelets were highly activated in KD patients (21, 34). Furthermore, Inositol-3-phosphate is known to be involved in platelet activation. There are reports showing that the single nucleotide variant of *inositol-trisphosphate 3-kinase C* (*ITPKC)* has the strongest impact on KD susceptibility and the treatment response (13, 35), and that *ITPKC* is involved in the activation of immune cells in patients with KD *via* inflammasome activation (19, 36). We directly examined the ability of platelets from KD patients to release Myl9 upon *in vitro* thrombin stimulation and found that the release of Myl9 from platelets of KD patients occurred independently of *in vitro* thrombin stimulation, indicating that platelets from KD patients are likely preactivated *in vivo*. Furthermore, in the LCWE-induced KD vasculitis mouse model, the expression of Myl9/12 was observed before inflammatory cells infiltrations. This result suggests that Myl9/12 is expressed in the very early phase of inflammation, and then CD69-positive cells are recruited and migrate into the vascular walls, thereby inducing vasculitis. These observations strongly suggest that plasma Myl9 and Myl9/12 expression in inflamed coronary artery of KD patients may potentially derive from activated platelets. However, alternatively, there is also a possibility that Myl9 may come from VSMCs. We found that Myl9/12 was highly detected in the arterial medial smooth muscle tissue in the vascular walls of control mice, in the absence of inflammation. LCWE-induced KD vasculitis resulted in the destruction of the three-layered artery, and Myl9/12 expression became weak and broad, supporting previous data showing that VSMCs become “synthetic” and downregulate the expression of contractile proteins, including Myl9 (19, 37). These data also raise a possibility that the broad expression of Myl9/12 may be derived from the destroyed vascular walls; however, we cannot draw any definitive conclusions at this time. In addition, we tried to quantify plasma Myl9 concentration in the control and LCWE-injected mice; however, the detection sensitivity was too low to detect murine plasma Myl9 by currently available methods. Therefore, we could not confirm the increase levels of Myl9 in LCWE-injected mice, which is a limitation of this study. Further studies are required to elucidate the detailed molecular mechanisms underlying how Myl9 is released into inflammatory sites and how it contributes to the pathogenesis of KD.

In this study, tissue samples obtained at autopsy from three KD patients were examined: in one case, the patient died in the acute phase of the disease (14 days after the onset), while the other two patients died in the chronic phase (1 year and 2 years after the onset, respectively). All patients were diagnosed with myocardial infarction. We found that, in the acute phase, Myl9/12 was most strongly expressed in the thrombus, especially in the white thrombus, which is mainly composed of platelets, and it was then strongly expressed in the intima and adventitia of the vessels, as well as the adventitia-side interstitial tissues. Interestingly, a previous histopathological report on KD at around 10 days of illness showed that neutrophils invade the blood vessel wall both from the intima and the adventitia side (38), which is the same orientation as the Myl9/12 expression that we detected in the autopsy specimen from the patient with acute-phase KD, suggesting that the Myl9/12 expression shows a strong association with inflammatory cell infiltration. In the chronic phase, the expression of Myl9/12 in the thrombus weakened and moved from the intima to the adventitia side. It is unclear whether changes in the Myl9/12 expression in the vessel wall are associated with vascular remodeling in the chronic phase.

One of the important findings of this study is that plasma Myl9 levels in KD patients were substantially high in the acute phase and decreased as the patient responded successfully to IVIG and recovered, indicating that plasma Myl9 levels reflect the disease activity of KD as measured by inflammatory markers. In fact, the plasma Myl9 levels were correlated with the expression levels of markers of KD disease activity, including CRP and the WBC and platelet counts. The plasma Myl9 levels of KD patients are much higher than those in other febrile illnesses, such as Epstein-Barr virus infection, adenovirus infection, cervical lymphadenitis, and *Yersinia* enteritis, the clinical features of which are similar to and often challenging to differentiate from KD. Importantly, blood Myl9 levels did not decrease in children who were IVIG non-responders, suggesting that plasma Myl9 levels may be considered as a biomarker for IVIG responsiveness. Plasma Myl9 levels may potentially also be used as a biomarker for other types of vasculitis. Indeed, we have recently reported that plasma Myl9 levels were elevated and correlated with disease severity and outcome in COVID-19 patients (39). SARS-CoV2 is known to infect cells *via* ACE2 receptors, which are expressed in blood vessels, and COVID-19 has been associated with vasculitis (40, 41). We found that plasma Myl9 levels in COVID-19 patients were similar to those in KD patients. These data suggest that plasma Myl9 levels may be elevated in different kinds of vasculitis, although further studies are required to make a definitive conclusion.

In conclusion, we demonstrated that Myl9 is highly detected in the plasma of KD patients, that Myl9/12 is abundantly detected in the inflamed coronary artery, and that the platelets of KD patients show abnormal characteristics. Thus, Myl9 could be a good biomarker for estimating the disease activity of KD and IVIG responsiveness. Further studies are needed to evaluate whether Myl9 indeed plays an important role in the pathogenesis of KD and it can be a therapeutic target of KD vasculitis.

## Data availability statement

The original contributions presented in the study are included in the article/**Supplementary Material**. Further inquiries can be directed to the corresponding authors.

## Ethics statement

The studies involving human participants were reviewed and approved by the Ethics Committee of Chiba University Graduate School of Medicine and Tokyo Women's Medical University Yachiyo Medical Center. Written informed consent to participate in this study was provided by the participants’ legal guardian/next of kin. The animal study was reviewed and approved by Chiba University Review Board for Animal Care.

## Author contributions

HK, HH, and MYK conceived the project and designed the experiments. HK, IH, YI, KA, TI, and MYK performed the experiments. YI, KA, NS, HI, and MZ provided human sample. ES, YL, MNR, and MA provided LCWE. YS and YKa helped perform the statistical analysis. ES, RE, YKu, YL, MNR, MA, and TN provided helpful advice. HK, HH, MYK, and TN wrote the manuscript with input from all authors. All authors contributed to the article and approved the submitted version.

## Supplementary material

Supplementary Figure 1 Plasma Myl9 levels of KD patients with CAA. **(A)** Plasma Myl9 levels at the acute phase (*i.e.* before first IVIG treatment) of IVIG responders (n=36) and non-responders (n=18). ns, not significant. Data are shown as the mean ± SEM. **(B)** Plasma Myl9 levels at the acute phase of KD patients with and without CAAs (n=76). CAAs were defined by a Z score of ≥2.5. ns, not significant. Data are shown as the mean ± SEM. **(C)** Plasma Myl9 levels at the acute phase of KD patients who did not respond to first IVIG treatment with and without CAAs (n=40). **(D)** Correlation between the plasma Myl9 levels at the acute phase and coronary artery diameter in each KD patient (n=76). **(E)** Plasma Myl9 levels at the acute phase in KD patients grouped based on the Z scores of coronary artery (Z scores: < 2.5, ≥ 2.5, ≥ 5, and ≥ 10) (n=76). ns, not significant. Data are shown as the mean ± SEM.

Supplementary Figure 2 Flow cytometric analysis of PBMCs from KD patients. **(A)** Gating strategy used to identify B cells (CD3^–^CD19^+^), CD4^+^ T cells (CD3^+^CD19^–^CD8^–^ CD4^+^) and CD8^+^ T cells (CD3^+^CD19^–^CD4^–^CD8^+^) in PBMCs from acute KD patients and HV children (Tube #1). Gating strategy used to identify NK cells including non-classical monocytes (CD16^+^CD14^–^), intermediate/non-classical monocytes (CD16^+^CD14^+^), and classical monocytes (CD16^–^CD14^+^) in PBMCs from acute KD patients and HV children (Tube #2). **(B)** Percentage of CD69 positive cells gated in A in KD patient and healthy volunteer (child). **(C)** Frequencies of CD69-expressing cells in NK, classical and non-classical/intermediate monocytes, B cells, CD4 and CD8 T cells in PBMCs from KD patients (IVIG responder) (n=6) in the acute phase and convalescent phase (1 month after IVIG treatment), and in child HVs (n=3). #*P* < 0.05 (single comparisons, Wilcoxon signed-rank test without correction). ns, not significant. Data are shown as the mean ± SEM.

Supplementary Figure 3 The cell number of CD69-positive PBMCs from KD patients. **(A)** The cell number of CD69-positive PBMCs of KD patients in the acute and convalescent phase (n=6) and HV (n=3). The same patient samples are connected by a line. Data are shown as the mean ± SEM. **(B)** The cell number of white blood cell (WBC, left), neutrophil (Neu, middle), and lymphocyte (Lym, right) of KD patients in the acute and convalescent phase (n=6) and HV (n=3). The same patient samples are connected by a line. Data are shown as the mean ± SEM.

Supplementary Figure 4 Flow cytometric analysis of peripheral bloods from LCWE-injected mice. **(A)** Flowcytometry analysis showing the various cells in the blood from the control mice (n=5) or LCWE-injected mice (day 7) (n=5). We identified B cells as B220 ^+^ CD3ε ^–^ cells, T cells as B220^–^CD3ε^+^ cells, NK cells as B220^–^CD3ε^–^NK1.1^+^ cells, Neutrophils as B220^–^CD3ε^–^CD11b^+^Ly6G^+^ cells, Eosinophils as B220^–^CD3ε^–^CD11b^+^Ly6G ^–^Ly6C^+^SSC^hi^ cells, Ly6C^+^ or Ly6C^–^ Monocytes as B220^–^CD3ε^–^CD11b^+^Ly6G ^–^SSC^low^ cells. Data are shown as the mean ± SEM. **(B)** The graph showing the frequency (left) and the absolute numbers in 100ml of peripheral blood (right) of each cells that identified in **(A)**. **(C)** The graph showing the frequency (left) and the absolute numbers in 100μl of peripheral blood (right) of CD69 positive each cells that identified in **(A)**. Data are shown as the mean ± SEM.

## References

[1] Burns JC Glodé MP. Kawasaki Syndrome. Lancet (2004) 364(9433):533–44. doi: 10.1016/s0140-6736(04)16814-1 15302199

[2] McCrindle BW Rowley AH Newburger JW Burns JC Bolger AF Gewitz M. Diagnosis, treatment, and long-term management of Kawasaki disease: A scientific statement for health professionals from the American heart association. Circulation (2017) 135(17):e927–e99. doi: 10.1161/CIR.0000000000000484 28356445

[3] Fukazawa R Kobayashi J Ayusawa M Hamada H Miura M Mitani Y. JCS/JSCS 2020 guideline on diagnosis and management of cardiovascular sequelae in Kawasaki disease. Circ J (2020) 84(8):1348–407. doi: 10.1253/circj.CJ-19-1094 32641591

[4] Newburger JW Takahashi M Burns JC Beiser AS Chung KJ Duffy CE. The treatment of Kawasaki syndrome with intravenous gamma globulin. N Engl J Med (1986) 315(6):341–7. doi: 10.1056/nejm198608073150601 2426590

[5] Burns JC Franco A. The immunomodulatory effects of intravenous immunoglobulin therapy in Kawasaki disease. Expert Rev Clin Immunol (2015) 11(7):819–25. doi: 10.1586/1744666x.2015.1044980 PMC4985263 26099344

[6] Hsieh LE Song J Tremoulet AH Burns JC Franco A. Intravenous immunoglobulin induces IgG internalization by tolerogenic myeloid dendritic cells that secrete IL-10 and expand fc-specific regulatory T cells. Clin Exp Immunol (2022) 208(3):361–71. doi: 10.1093/cei/uxac046 PMC9226148 35536993

[7] Hamada H Suzuki H Abe J Suzuki Y Suenaga T Takeuchi T. Inflammatory cytokine profiles during cyclosporin treatment for immunoglobulin-resistant Kawasaki disease. Cytokine (2012) 60(3):681–5. doi: 10.1016/j.cyto.2012.08.006 22944461

[8] Wang Y Wang W Gong F Fu S Zhang Q Hu J. Evaluation of intravenous immunoglobulin resistance and coronary artery lesions in relation to Th1/Th2 cytokine profiles in patients with Kawasaki disease. Arthritis Rheum (2013) 65(3):805–14. doi: 10.1002/art.37815 23440694

[9] Ghosh P Katkar GD Shimizu C Kim J Khandelwal S Tremoulet AH. An artificial intelligence-guided signature reveals the shared host immune response in MIS-c and Kawasaki disease. Nat Commun (2022) 13(1):2687. doi: 10.1038/s41467-022-30357-w 35577777 PMC9110726

[10] Rowley AH Shulman ST. Kawasaki Syndrome. Clin Microbiol Rev (1998) 11(3):405–14. doi: 10.1128/cmr.11.3.405 PMC88887 9665974

[11] Onouchi Y Gunji T Burns JC Shimizu C Newburger JW Yashiro M. ITPKC functional polymorphism associated with Kawasaki disease susceptibility and formation of coronary artery aneurysms. Nat Genet (2008) 40(1):35–42. doi: 10.1038/ng.2007.59 18084290 PMC2876982

[12] Onouchi Y Suzuki Y Suzuki H Terai M Yasukawa K Hamada H. ITPKC and CASP3 polymorphisms and risks for IVIG unresponsiveness and coronary artery lesion formation in Kawasaki disease. Pharmacogenomics J (2013) 13(1):52–9. doi: 10.1038/tpj.2011.45 21987091

[13] Hamada H Suzuki H Onouchi Y Ebata R Terai M Fuse S. Efficacy of primary treatment with immunoglobulin plus ciclosporin for prevention of coronary artery abnormalities in patients with Kawasaki disease predicted to be at increased risk of non-response to intravenous immunoglobulin (KAICA): a randomised controlled, open-label, blinded-endpoints, phase 3 trial. Lancet (2019) 393(10176):1128–37. doi: 10.1016/s0140-6736(18)32003-8 30853151

[14] Kimura MY Hayashizaki K Tokoyoda K Takamura S Motohashi S Nakayama T. Crucial role for CD69 in allergic inflammatory responses: CD69-Myl9 system in the pathogenesis of airway inflammation. Immunol Rev (2017) 278(1):87–100. doi: 10.1111/imr.12559 28658550

[15] Kimura MY Koyama-Nasu R Yagi R Nakayama T. A new therapeutic target: the CD69-Myl9 system in immune responses. Semin Immunopathol (2019) 41(3):349–58. doi: 10.1007/s00281-019-00734-7 30953160

[16] Miki-Hosokawa T Hasegawa A Iwamura C Shinoda K Tofukuji S Watanabe Y. CD69 controls the pathogenesis of allergic airway inflammation. J Immunol (2009) 183(12):8203–15. doi: 10.4049/jimmunol.0900646 19923457

[17] Hayashizaki K Kimura MY Tokoyoda K Hosokawa H Shinoda K Hirahara K. Myosin light chains 9 and 12 are functional ligands for CD69 that regulate airway inflammation. Sci Immunol (2016) 1(3):eaaf9154. doi: 10.1126/sciimmunol.aaf9154 28783682

[18] Yokoyama M Kimura MY Ito T Hayashizaki K Endo Y Wang Y. Myosin light chain 9/12 regulates the pathogenesis of inflammatory bowel disease. Front Immunol (2020) 11:594297. doi: 10.3389/fimmu.2020.594297 33584659 PMC7878395

[19] Noval Rivas M Wakita D Franklin MK Carvalho TT Abolhesn A Gomez AC. Intestinal permeability and IgA provoke immune vasculitis linked to cardiovascular inflammation. Immunity (2019) 51(3):508–21.e6. doi: 10.1016/j.immuni.2019.05.021 31471109 PMC6751009

[20] Taki M Kobayashi M Ohi C Shimizu H Goto K Aso K. Spontaneous platelet aggregation in Kawasaki disease using the particle counting method. Pediatr Int (2003) 45(6):649–52. doi: 10.1111/j.1442-200x.2003.01810.x 14651534

[21] Yahata T Suzuki C Yoshioka A Hamaoka A Ikeda K. Platelet activation dynamics evaluated using platelet-derived microparticles in Kawasaki disease. Circ J (2014) 78(1):188–93. doi: 10.1253/circj.cj-12-1037 24152721

[22] Kim HJ Choi EH Lim YJ Kil HR. The usefulness of platelet-derived microparticle as biomarker of antiplatelet therapy in Kawasaki disease. J Korean Med Sci (2017) 32(7):1147–53. doi: 10.3346/jkms.2017.32.7.1147 PMC5461319 28581272

[23] Zheng X Wu W Zhang Y Wu G. Changes in and significance of platelet function and parameters in Kawasaki disease. Sci Rep (2019) 9(1):17641. doi: 10.1038/s41598-019-54113-1 31776411 PMC6881449

[24] Kobayashi T Inoue Y Takeuchi K Okada Y Tamura K Tomomasa T. Prediction of intravenous immunoglobulin unresponsiveness in patients with Kawasaki disease. Circulation (2006) 113(22):2606–12. doi: 10.1161/CIRCULATIONAHA.105.592865 16735679

[25] Kobayashi T Ayusawa M Suzuki H Abe J Ito S Kato T. Revision of diagnostic guidelines for Kawasaki disease (6th revised edition). Pediatr Int (2020) 62(10):1135–8. doi: 10.1111/ped.14326 33001522

[26] Shulman ST Rowley AH. Kawasaki Disease: insights into pathogenesis and approaches to treatment. Nat Rev Rheumatol (2015) 11(8):475–82. doi: 10.1038/nrrheum.2015.54 25907703

[27] Kobayashi T Fuse S Sakamoto N Mikami M Ogawa S Hamaoka K. A new z score curve of the coronary arterial internal diameter using the lambda-Mu-Sigma method in a pediatric population. J Am Soc Echocardiogr (2016) 29(8):794–801.e29. doi: 10.1016/j.echo.2016.03.017 27288089

[28] Lee Y Schulte DJ Shimada K Chen S Crother TR Chiba N. Interleukin-1β is crucial for the induction of coronary artery inflammation in a mouse model of Kawasaki disease. Circulation (2012) 125(12):1542–50. doi: 10.1161/circulationaha.111.072769 PMC3337219 22361326

[29] Suganuma E Sato S Honda S Nakazawa A. A novel mouse model of coronary stenosis mimicking Kawasaki disease induced by lactobacillus casei cell wall extract. Exp Anim (2020) 69(2):233–41. doi: 10.1538/expanim.19-0124 PMC7220718 31932543

[30] Collins TJ. ImageJ for microscopy. Biotechniques (2007) 43(1 Suppl):25–30. doi: 10.2144/000112517 17936939

[31] Elzey BD Tian J Jensen RJ Swanson AK Lees JR Lentz SR. Platelet-mediated modulation of adaptive immunity. Immunity (2003) 19(1):9–19. doi: 10.1016/s1074-7613(03)00177-8 12871635

[32] Ae R Abrams JY Maddox RA Schonberger LB Nakamura Y Shindo A. Platelet count variation and risk for coronary artery abnormalities in Kawasaki disease. Pediatr Infect Dis J (2020) 39(3):197–203. doi: 10.1097/inf.0000000000002563 31851145

[33] Ikeda K Yamaguchi K Tanaka T Mizuno Y Hijikata A Ohara O. Unique activation status of peripheral blood mononuclear cells at acute phase of Kawasaki disease. Clin Exp Immunol (2010) 160(2):246–55. doi: 10.1111/j.1365-2249.2009.04073.x PMC2857948 20015095

[34] Suzuki C Yahata T Okamoto-Hamaoka A Fujii M Yoshioka A Niwa Y. Utility of whole-blood aggregometry for evaluating anti-platelet therapy for Kawasaki disease. Pediatr Int (2013) 55(5):550–4. doi: 10.1111/ped.12120 23659651

[35] Onouchi Y Ozaki K Burns JC Shimizu C Terai M Hamada H. A genome-wide association study identifies three new risk loci for Kawasaki disease. Nat Genet (2012) 44(5):517–21. doi: 10.1038/ng.2220 22446962

[36] Alphonse MP Duong TT Shumitzu C Hoang TL McCrindle BW Franco A. Inositol-triphosphate 3-kinase c mediates inflammasome activation and treatment response in Kawasaki disease. J Immunol (2016) 197(9):3481–9. doi: 10.4049/jimmunol.1600388 27694492

[37] Porritt RA Zemmour D Abe M Lee Y Narayanan M Carvalho TT. NLRP3 inflammasome mediates immune-stromal interactions in vasculitis. Circ Res (2021) 129(9):e183–200. doi: 10.1161/circresaha.121.319153 PMC8555446 34517723

[38] Takahashi K Oharaseki T Naoe S Wakayama M Yokouchi Y. Neutrophilic involvement in the damage to coronary arteries in acute stage of Kawasaki disease. Pediatr Int (2005) 47(3):305–10. doi: 10.1111/j.1442-200x.2005.02049.x 15910456

[39] Iwamura C Hirahara K Kiuchi M Ikehara S Azuma K Shimada T. Elevated Myl9 reflects the Myl9-containing microthrombi in SARS-CoV-2-induced lung exudative vasculitis and predicts COVID-19 severity. Proc Natl Acad Sci U.S.A. (2022) 119(33):e2203437119. doi: 10.1073/pnas.2203437119 35895716 PMC9388124

[40] McGonagle D Bridgewood C Ramanan AV Meaney JFM Watad A. COVID-19 vasculitis and novel vasculitis mimics. Lancet Rheumatol (2021) 3(3):e224–e33. doi: 10.1016/s2665-9913(20)30420-3 PMC7832717 33521655

[41] Nicosia RF Ligresti G Caporarello N Akilesh S Ribatti D. COVID-19 vasculopathy: Mounting evidence for an indirect mechanism of endothelial injury. Am J Pathol (2021) 191(8):1374–84. doi: 10.1016/j.ajpath.2021.05.007 PMC8141344 34033751

