# Supplementary figures and images for: Increased Myosin light chain 9 expression during Kawasaki disease vasculitis
Source: Front Immunol. 2023 Jan 6;13:1036672. doi: 10.3389/fimmu.2022.1036672 (PMC9853906; doi:10.3389/fimmu.2022.1036672)

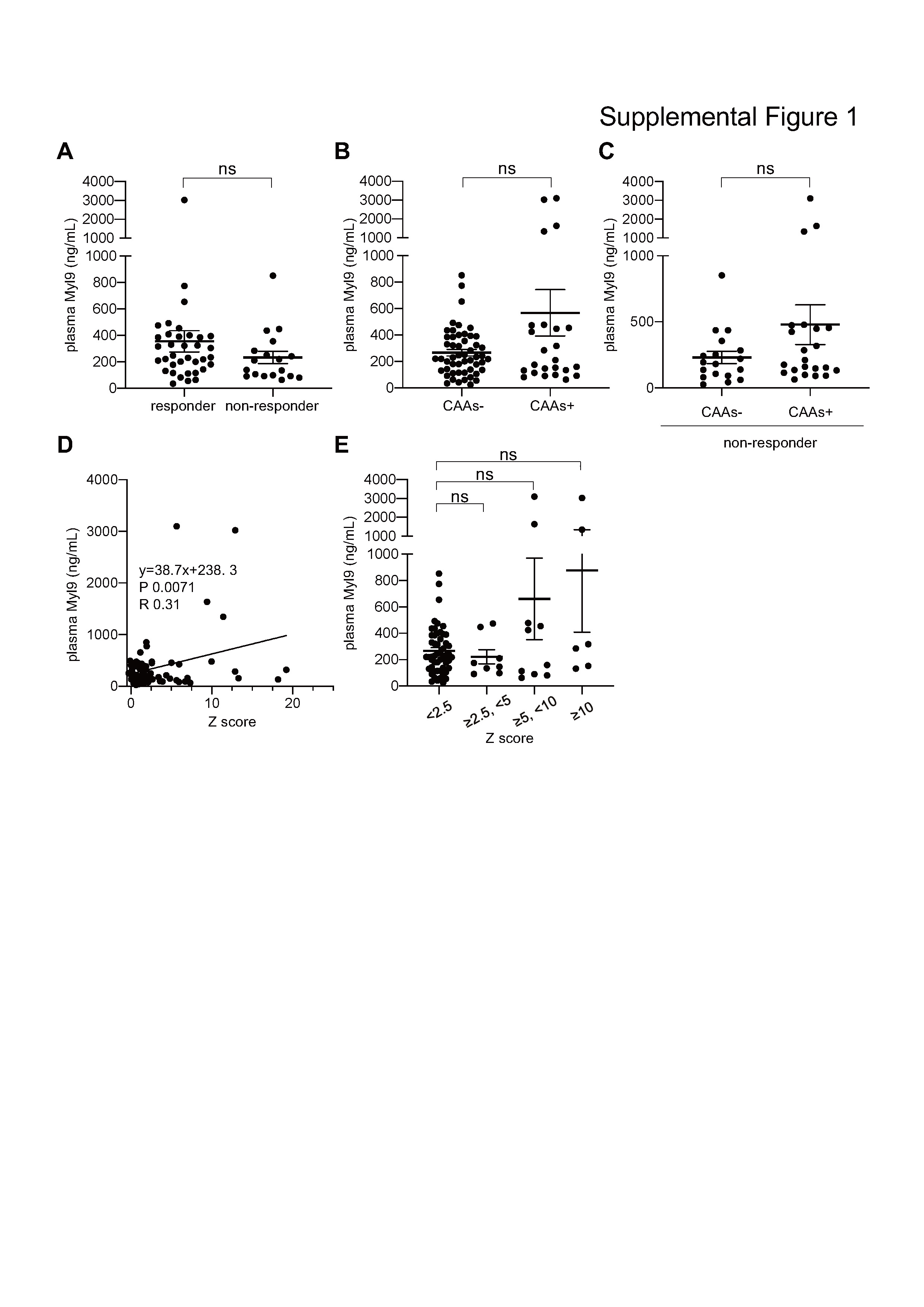

Supplement: Supplementary Figure 1 — Plasma Myl9 levels of KD patients with CAA. (A) Plasma Myl9 levels at the acute phase (i.e. before first IVIG treatment) of IVIG responders (n=36) and non-responders (n=18). ns, not significant. Data are shown as the mean ± SEM. (B) Plasma Myl9 levels at the acute phase of KD patients with and without CAAs (n=76). CAAs were defined by a Z score of ≥2.5. ns, not significant. Data are shown as the mean ± SEM. (C) Plasma Myl9 levels at the acute phase of KD patients who did not respond to first IVIG treatment with and without CAAs (n=40). (D) Correlation between the plasma Myl9 levels at the acute phase and coronary artery diameter in each KD patient (n=76). (E) Plasma Myl9 levels at the acute phase in KD patients grouped based on the Z scores of coronary artery (Z scores: < 2.5, ≥ 2.5, ≥ 5, and ≥ 10) (n=76). ns, not significant. Data are shown as the mean ± SEM. [file Image_1.jpg]

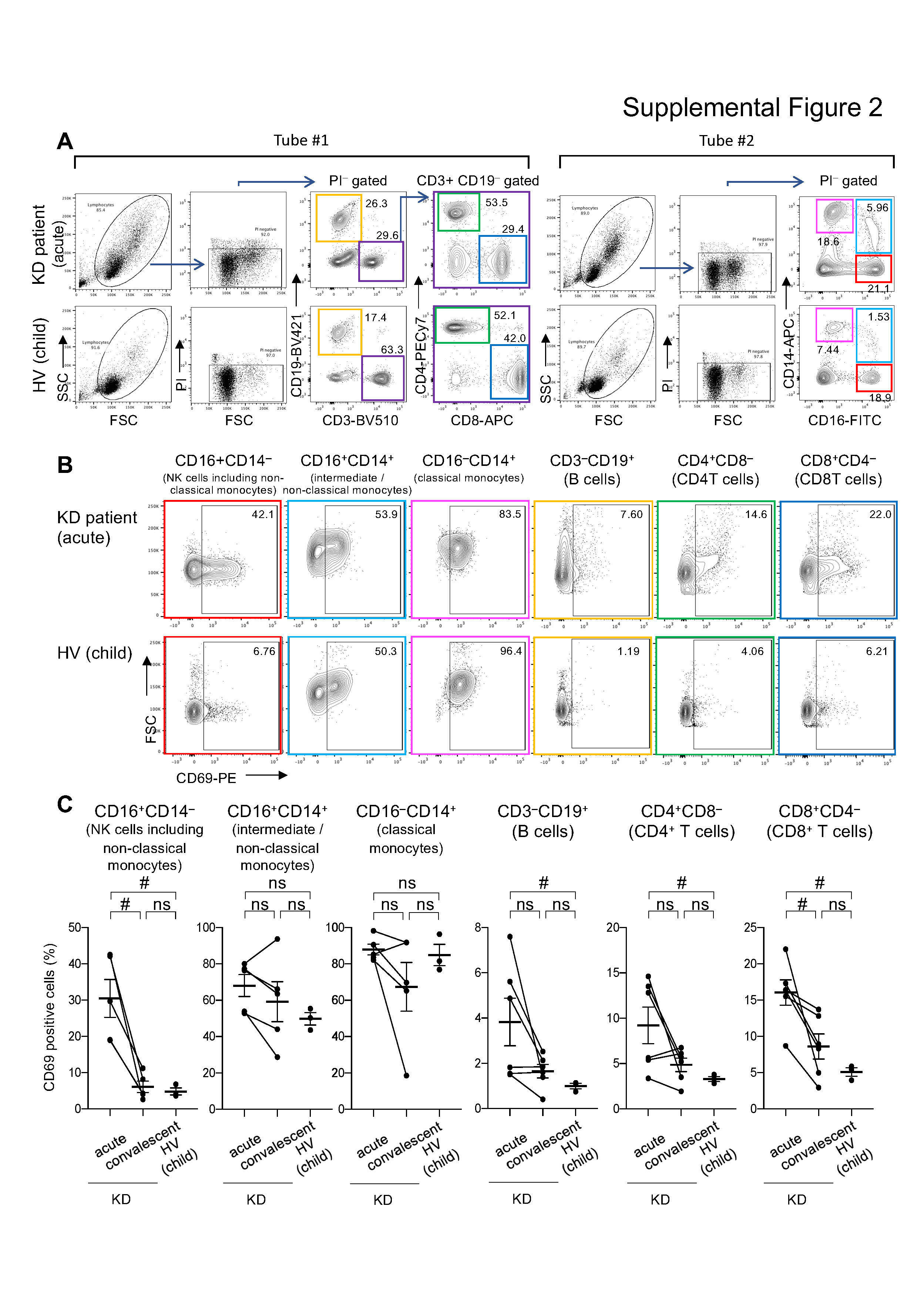

Supplement: Supplementary Figure 2 — Flow cytometric analysis of PBMCs from KD patients. (A) Gating strategy used to identify B cells (CD3–CD19+), CD4+ T cells (CD3+CD19–CD8– CD4+) and CD8+ T cells (CD3+CD19–CD4–CD8+) in PBMCs from acute KD patients and HV children (Tube #1). Gating strategy used to identify NK cells including non-classical monocytes (CD16+CD14–), intermediate/non-classical monocytes (CD16+CD14+), and classical monocytes (CD16–CD14+) in PBMCs from acute KD patients and HV children (Tube #2). (B) Percentage of CD69 positive cells gated in A in KD patient and healthy volunteer (child). (C) Frequencies of CD69-expressing cells in NK, classical and non-classical/intermediate monocytes, B cells, CD4 and CD8 T cells in PBMCs from KD patients (IVIG responder) (n=6) in the acute phase and convalescent phase (1 month after IVIG treatment), and in child HVs (n=3). #P < 0.05 (single comparisons, Wilcoxon signed-rank test without correction). ns, not significant. Data are shown as the mean ± SEM. [file Image_2.jpg]

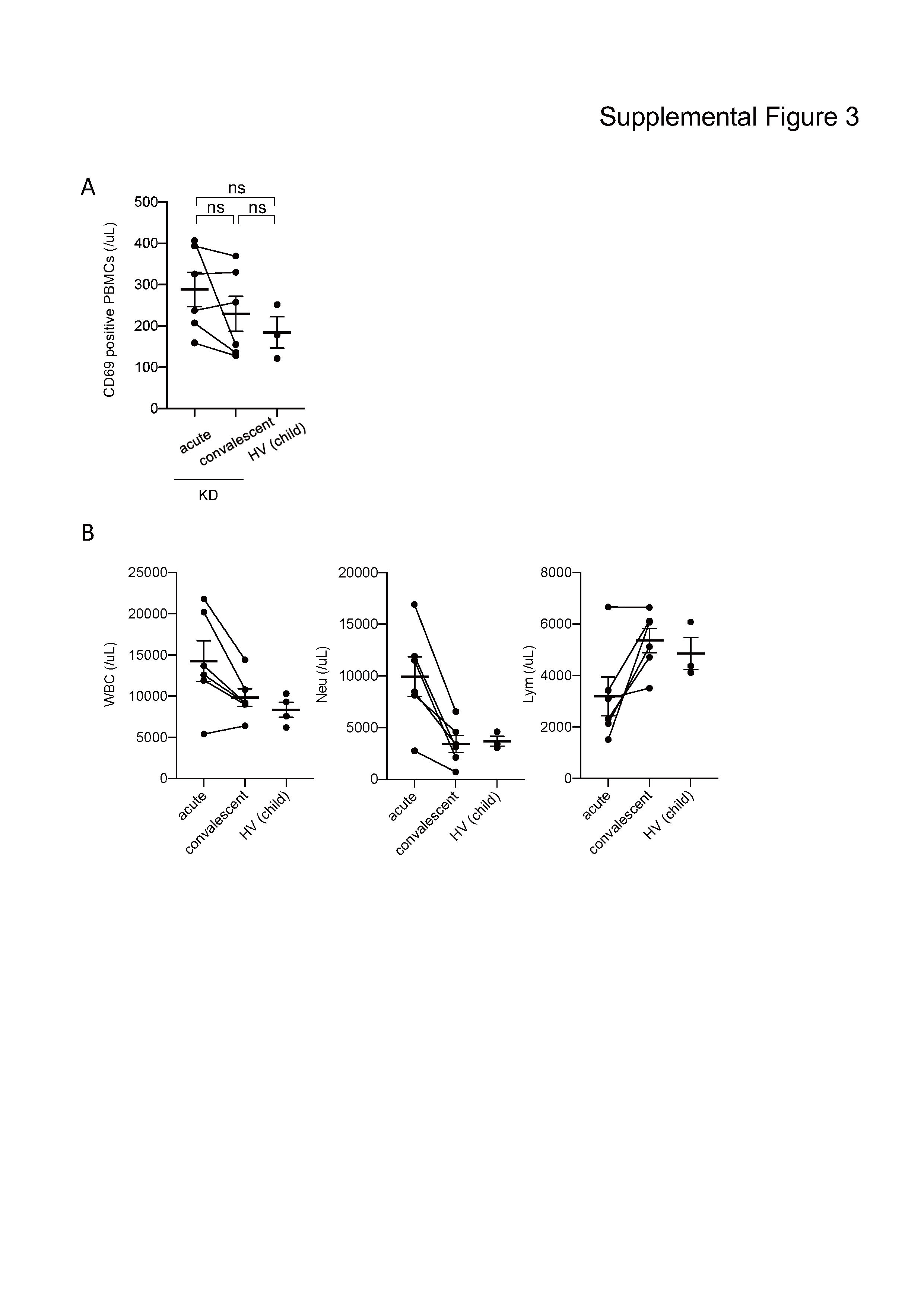

Supplement: Supplementary Figure 3 — The cell number of CD69-positive PBMCs from KD patients. (A) The cell number of CD69-positive PBMCs of KD patients in the acute and convalescent phase (n=6) and HV (n=3). The same patient samples are connected by a line. Data are shown as the mean ± SEM. (B) The cell number of white blood cell (WBC, left), neutrophil (Neu, middle), and lymphocyte (Lym, right) of KD patients in the acute and convalescent phase (n=6) and HV (n=3). The same patient samples are connected by a line. Data are shown as the mean ± SEM. [file Image_3.jpg]

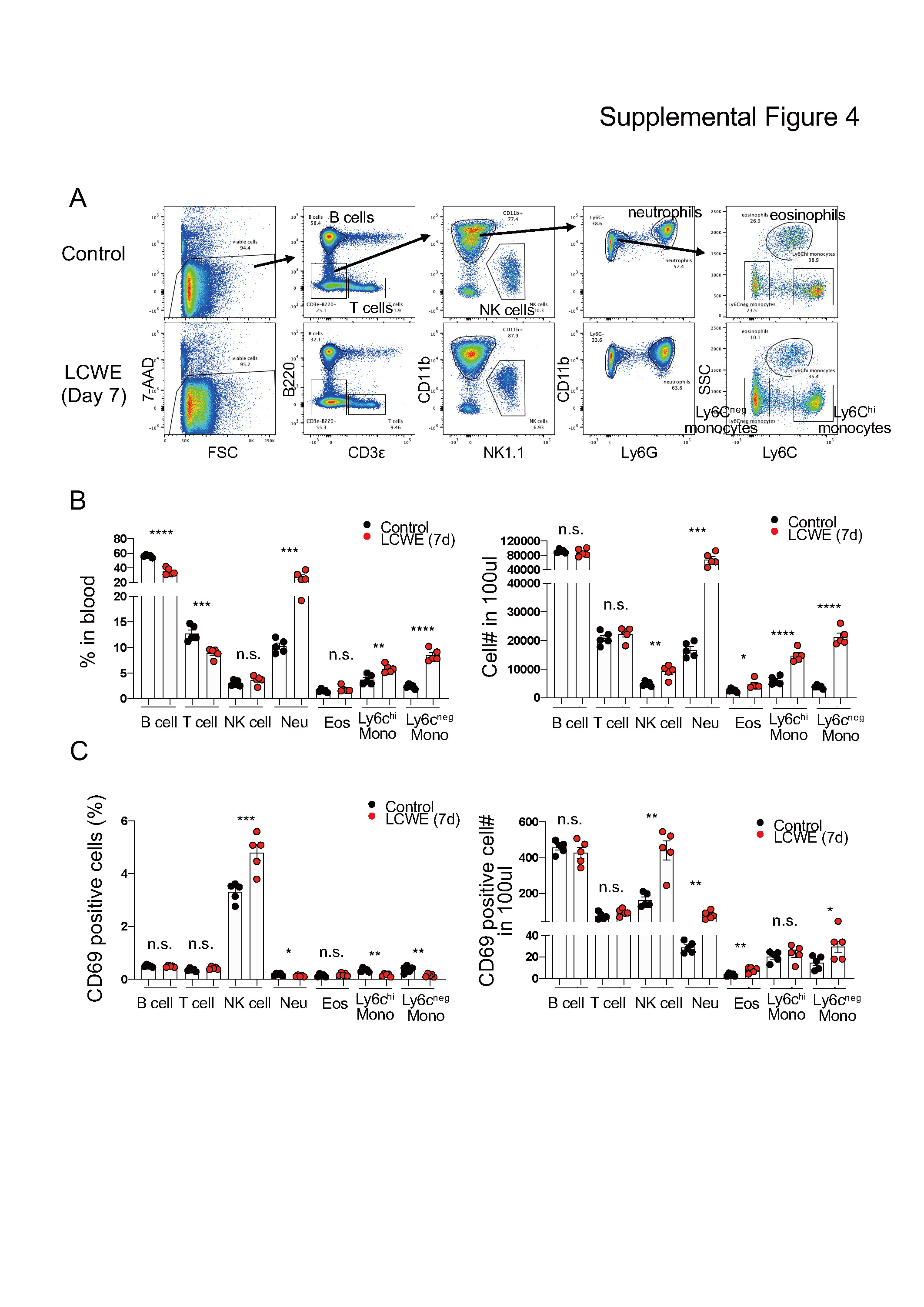

Supplement: Supplementary Figure 4 — Flow cytometric analysis of peripheral bloods from LCWE-injected mice. (A) Flowcytometry analysis showing the various cells in the blood from the control mice (n=5) or LCWE-injected mice (day 7) (n=5). We identified B cells as B220 + CD3ε – cells, T cells as B220–CD3ε+ cells, NK cells as B220–CD3ε–NK1.1+ cells, Neutrophils as B220–CD3ε–CD11b+Ly6G+ cells, Eosinophils as B220–CD3ε–CD11b+Ly6G –Ly6C+SSChi cells, Ly6C+ or Ly6C– Monocytes as B220–CD3ε–CD11b+Ly6G –SSClow cells. Data are shown as the mean ± SEM. (B) The graph showing the frequency (left) and the absolute numbers in 100ml of peripheral blood (right) of each cells that identified in (A). (C) The graph showing the frequency (left) and the absolute numbers in 100μl of peripheral blood (right) of CD69 positive each cells that identified in (A). Data are shown as the mean ± SEM. [file Image_4.jpg]
